# Exopolysaccharide Produced by *Lactobacillus Plantarum* Induces Maturation of Dendritic Cells in BALB/c Mice

**DOI:** 10.1371/journal.pone.0143743

**Published:** 2015-11-24

**Authors:** Yanjun Tang, Wei Dong, Keyu Wan, Ligang Zhang, Chun Li, Lili Zhang, Ning Liu

**Affiliations:** 1 Key Laboratory of Dairy Science, Ministry of Education; College of Food Science, Northeast Agricultural University, Harbin, China; 2 National Dairy Engineering & Research Center, Harbin, China; 3 Synergetic Innovation Center of Food Safety and Nutrition, Harbin, China; 4 College of Food Science, Heilongjiang Bayi Agricultural University, Daqing, China; Institut National de la Santé et de la Recherche Médicale (INSERM), FRANCE

## Abstract

*Lactobacillus plantarum* (*L*. *plantarum*) exopolysaccharide (EPS) is an important bioactive component in fermented functional foods. However, there is a lack of data concerning the effects of *L*. *plantarum* EPS on maturation of mouse dendritic cells (DCs). In this study, we purified *L*. *plantarum* EPS and examined its effects on cytokines production by dendritic cells in serum and intestinal fluid of BALB/c mice, then investigated its effects on phenotypic and functional maturation of mouse bone marrow-derived dendritic cells (BMDCs). Cytokines (nitric oxide, IL-12p70, IL-10 and RANTES) in serum and intestinal fluid were analyzed by enzyme linked immunosorbent assay (ELISA) after the mice received EPS for 2, 5 and 7 days, respectively. DCs derived from bone marrow of BALB/c mouse were treated with EPS, then the phenotypic maturation of BMDCs was analyzed using flow cytometer and the functional maturation of BMDCs was analyzed by ELISA, and, lastly, mixed lymphocyte proliferation was performed. We found the molecular weight of purified EPS was approximately 2.4×10^6^ Da and it was composed of ribose, rhamnose, arabinose, xylose, mannose, glucose and galactose in a molar ratio of 2:1:1:10:4:205:215. We observed that *L*. *plantarum* EPS enriched production of nitric oxide, IL-12p70 and RANTES, and decreased the secretion of IL-10 in the serum or intestinal fluid as well as in the supernatant of DCs treated with the EPS. The EPS also up-regulated the expression of MHC II and CD86 on DCs surface and promoted T cells to proliferate *in vitro*. Our data provide direct evidence to suggest that *L*. *plantarum* EPS can effectively induce maturation of DCs in mice.

## Introduction

*Lactobacillus plantarum (L*. *plantarum)*, a Gram-positive bacteria commonly found in nature, has industrial importance as a key component of fermenters used in probiotic fermented milk products, especially in sausage and cheese, which are consumed by an increasing number of people worldwide [1]. The potential benefits of *L*. *plantarum* as a probiotic for human health include regulating the immune system, reducing serum cholesterol, keeping intestinal flora balance, and reducing the risk of tumors [1]. Strains of *L*. *plantarum* have proven to produce lactic acid and bactericidal bioactive compounds, enabling them to colonize the human intestinal tract, and express their antagonistic potential against intestinal pathogens [2,3]. One of the most important bioactive compounds produced by *L*. *plantarum* is exopolysaccharide (EPS).

EPS, secreted by lactic acid bacteria (LAB), is a long-chain polysaccharide consisting of glucose, galactose, fructose, mannose or other monosaccharides [4]. EPS from LAB can be applied to improve the texture, rheology and taste of fermented milk products such as yoghurt, cheese, viili and långfil [4,5]. In recent years, some researchers have reported LAB exopolysaccharides for their antibiofilm, antioxidant, antitumour, and immunostimulatory activities [6–9].

*L*. *plantarum* EPS can interact dynamically over extended periods of time with intestinal mucosa, which presents the greatest surface of interchange between the human body and the external environment. The mucosa’s intestinal microflora, mucosal barrier and mucosal immune system can protect the host from harmful compounds it ingests, such as pathogens [10]. Some studies have shown that administration of probiotic fermented milk for a long time could increase dendritic cell numbers in the intestinal mucosal immune system [11,12]. As an important bioactive component of fermented milk products, EPS of LAB may play a crucial role within the intestinal mucosal immune system.

DCs, derived from bone marrow monocytes and can be seeded in intestine, are professional antigen-presenting cells which can initiate immune responses of intestinal mucosa. DCs in the intestinal lamina propria (LP) are able to open the junctions between adjacent epithelial cells to send dendrites out to capture bacteria or proteins from the intestinal lumen [13]. CX3C-chemokine receptor (CX3CR)1 on DCs is indispensable for formation of transepithelial dendrite, and the dendrites found especially in the terminal ileum, where a great amount of CX3C-chemokine ligand (CX3CL)1 is expressed [14,15]. Thus, when mice are deficient for CX3CR1, LP DC dendrites cannot extend into the lumen followed by failing to sample *Salmonella* resulting in unprotecting the mucosa from pathogenic bacteria [15]. LP DCs can sample invasive and noninvasive bacteria by their transepithelial dendrites [13,15], which suggests a possible mechanism for LP DCs in presenting pathogenic antigens to naive T cells in draining mesenteric lymph nodes (MLNs). The sampling mechanism also occurs in Peyer’s patches (PPs) and includes interaction of M cells in the follicle-associated epithelium and DCs in the subepithelial dome [16]. These studies show that DCs can be exposed to food ingredients in the lumen of the small intestine. Thus, the EPS from *L*. *plantarum* in fermented dairy products may interact with the DCs in the lumen of the small intestine. Some polysaccharides have been reported for stimulating the maturation of murine bone-marrow-derived dendritic cells (BMDCs) *in vitro* [17,18]. However, at present there is no published article concerning whether *L*. *plantarum* EPS has the same immunoregulation on DCs *in vivo* and *in vitro*.

So the aim of our work was to investigate the modulation of *L*. *plantarum* EPS on maturation of DCs *in vivo* and *in vitro*. When DCs mature, they synthesize high levels of NO, interleukin (IL) 12, and RANTES, and express more costimulatory molecules such as CD86 and major histocompatibility complex (MHC), all of which can induce T cells to become fully activated to initial immune response [19]. In this study, *L*. *plantarum* EPS was purified and subsequently fed to mice by gavage to analyze the cytokines production in serum and intestinal fluid. To investigate the effect of EPS on DCs, BMDCs were treated with EPS, then phenotypic and functional maturation of BMDCs were analyzed. Finally the effect of *L*. *plantarum* EPS on allogeneic lymphocyte proliferation was investigated.

## Materials and Methods

### Ethics statement

Our study was carried out in accordance with the Guide for the Care and Use of Laboratory Animals of the National Institutes of Health (2011) and Guidelines for the Euthanasia of Animals of American Veterinary Medical Association (2013). The protocol was approved by the Committee on the Ethics of Animal Experiments of Northeast Agricultural University (Permit Number: 20130518–03). These animals were anesthetized by injection of sodium pentobarbital (50 mg/kg) to obtain blood from their hearts, and then sacrificed by cervical dislocation to remove their small intestines for the intestinal fluid. All efforts aimed to minimize suffering of the mice. No mouse became ill or died during the experiment.

### Chemicals

In this study, lipopolysaccharide (LPS), as positive control, was purchased from Sigma-Aldrich and RPMI 1640 complete medium (RPMI 1640) were sourced from Thermo Fisher Scientific Inc. (Gibco, US). The NO assay kits were products of Hangzhou Sijiqing Co. (Hangzhou, China). The fluorescent monoclonal antibodies (PE-CD11c, FITC-MHCII and FITC-CD86) were purchased from eBioscience (San Diego, CA) and the ELISA assay kits for IL-12p70, IL-10 and RANTES were also products of eBioscience. Other reagents were mainly from either Sigma-Aldrich or Invitrogen.

### Bacterial strains and culture conditions

*L*. *plantarum* was purchased from the Institute of Microbiology, Chinese Academy of Sciences, China. It was isolated from fermented foods and was identified by 16S rDNA sequence alignment. The medium used for fermentation by *L*. *plantarum* producing EPS consisted of 12% skim milk powder, 1% peptone (Oxoid), 1.5% glucose (Oxoid) and 0.1% K_2_HPO_4_. All of these ingredients were mixed in water and heat treated at 121°C for 15 min. Fermentation was carried out at 37°C for 20 hours with a 3% inoculum.

### Separation and purification of the EPS

After the incubation of *L*. *plantarum* for 20 hours, trichloroacetic acid was added to the cultures to a final concentration of 4% (w/v), and the cultures were stirred at 4°C for 12 h, then centrifuged at 10000 *g* for 20 min at 4°C. Finally, the supernatant was collected and concentrated to 25% of the original volume. EPS was precipitated by gradually adding an equal volume of cold ethanol and was collected by centrifugation, then diverted into dialysis bags (MW 7000 ~ 14000) which were immersed in deionized water. The water was refreshed once every 8 h for 3 d. Finally, the aqueous EPS solutions were freeze-dried in a DURA-DRY freeze-dryer and the EPS lyophilized powder was collected for later study. The content of total sugar in the powder was determined by a colorimetric method as previously described [20] and the absorbance was measured at 490 nm in a spectrophotometer. The concentration of protein was measured by the Bradford method using a protein assay kit following the manufacturer’s instructions. The content of endotoxin in the powder was determined using a chromogenic Limulus amedocyte lysate kit (Associates of Cape Cod, USA).

### Monosaccharide and molecular weight (Mw) determination for the EPS

The monosaccharides determination of *L*. *plantarum* EPS was carried out by gas chromatography (GC). The Mw of the polysaccharide was determined by size exclusion chromatography (SEC). GC and SEC analysis were performed referring to protocols reported in previous work [21]. Five dextrans standards (Mw 344.8, 606.2, 1185, 1907 and 2800kDa) were used to establish a linear regression for Mw determination.

### Mice and feeding

BALB/c female mice (6 ~ 8 weeks old, 18~22 g) were purchased from Charles River (Beijing, China). These animals were randomly divided into phosphate buffered saline (PBS) group, EPS group and LPS group and they were housed in plastic cages kept in a specific-pathogen-free (SPF) atmosphere with temperature 23± 2°C, humidity 55 ± 2% and a 12 h light/dark cycle. Mice management was in accordance with the guide for the care and use of laboratory animals of the National Institutes of Health.

*L*. *plantarum* EPS or LPS was dissolved in PBS solution. Tested mice received 100 mg/kg body weight/day of EPS by gavage for 2, 5 or 7 days, while positive control mice received 1 mg/kg body weight/day of LPS and negative control mice received PBS instead. All mice received a basic balanced diet and water ad libitum. After 2, 5 and 7 days of administration, these animals were anesthetized respectively by injection of sodium pentobarbital (50 mg/kg) to obtain blood from their hearts, then they were sacrificed by cervical dislocation to remove their small intestines for the intestinal fluid.

### Cytokine determination in serum and intestinal fluid

The blood was kept at 37°C for 1 h, and then centrifuged at 6000 *g* for 10 min at 4°C to obtain the blood serum. Mixture in the small intestine of the mice was flushed with PBS and it was centrifuged at 10,000 *g* for 10 min at 4°C to get supernatant. The supernatant and the serum were kept frozen at -80°C until use. NO activity was determined using NO assay kits (Nanjing Jiancheng Bioengineering Institute, Nanjing, China), and IL-12p70, IL-10 and RANTES were quantified by commercial mouse IL-12p70, IL-10 and RANTES enzyme-linked immunosorbent assay (ELISA) kits following the manufacturer’s instructions.

### Induction of BMDC

BALB/c mice were housed in SPF animal facilities with free access to water and food. The method used to isolate and cultivate BMDCs was adapted with slight modification from Inaba et al., 1992 [22]. Briefly, bone marrow cells were obtained from BALB/c mice by flushing tibias and femurs with RPMI 1640 complete medium (RPMI l640) (Gibco, Thermo Fisher Scientific Inc.) under aseptic conditions. After washing cells out of bone marrow, red blood cells were lysed by Tris ammonium chloride and bone marrow monocytes were cultured for 24 h in RPMI 1640 media containing 10% fetal bovine serum (FBS; Biological Engineering Materials Co., Ltd. Hangzhou Evergreen, China). After lysing and culturing, these cells were seeded into 24-well plates with 1.0 × 10^6^ cells in each well in 1 mL of RPMI 1640/10% FBS supplemented with recombinant (rm) murine granulocyte macrophage colony-stimulating factor (GM-CSF) (20 ng/ml) and rm IL-4 (20 ng/ml), and incubated at 37°C with 5% CO_2_ air. During a 6-day culture, nonadherent cells were discarded then rm GM-CSF and rm IL-4 were refreshed by replacing one-half of the supernatant volume with fresh medium every 2 days and collecting loosely attached cells as bone-marrow-derived dendritic cell for later use.

### The flow cytometry assay for DCs surface markers

DCs cultured for 6 days were divided into three groups. The EPS was added to a final concentration of 0 (RPMI 1640) and 100 μg/mL for negative control and experimental groups, respectively. LPS was added to a final concentration of 1 μg/mL for the positive control. DCs were kept at 37°C for 24 h and then washed twice in cold PBS buffer (containing 2% (v/v) bovine serum albumin in PBS). After washing, the cells were placed in a round-bottom 96-well plate supplemented with 10% (v/v) goat serum and allowed to interact for 10 min. Subsequently, the cells were centrifuged and resuspended in PBS buffer at a concentration of 1 × 10^6^/mL. The sample was incubated for 30 min after addition of PE-CD11c, FITC-CD86 and FITC-MHC II to a 100 μL cell suspension. All steps were conducted on ice and in the dark. Cells were washed twice in PBS buffer and resuspended in 100 μL PBS buffer respectively for flow cytometric analysis on an EPICS XL flow cytometer with FACSDiva software (Version 6.1) for analyze data analysis (Beckman Coulter Inc., USA).

### Determination of cytokine production

The loose adherent DCs were suspended with a sterile pipette and collected by centrifugation (300 *g*, 5 min), then were resuspended in RPMI 1640 with rm GM-CSF and rm IL-4. The cell concentration was adjusted to 1 × 10^5^/mL, and then the cell solution was transferred in a 24-well plate. EPS was added to each well with final concentrations of 0 (RPMI 1640, as negative control), 25, 50, 100, 150 and 200 μg/mL, and LPS was added to a final concentration of 1 μg/mL as positive control. The 24-well plate was incubated at 37°C and 5% CO_2_ for 24 h. The supernatant was collected and frozen at -80°C until use. NO, IL-12p70, IL-10 and RANTES in supernatant were determined by the corresponding kits or mouse ELISA Sets.

### Mixed lymphocyte reaction

After red blood cells were lysed by Tris-NH_4_Cl, BALB/c mouse spleen cells were resuspended and subsequently mixed with lymphocyte separation medium and RPMI l640, centrifuged at 300 *g* for 30 min. The lymphocyte was transferred to a 24-well plate and mixed with the BMDCs, which were treated with 0 (RPMI 1640, as negative control), 50, and 200 μg/mL EPS, and 1 μg/mL LPS (as positive control), respectively. After mixing, the cells were inactivated by mitomycin C, then incubated at 37°C for 72 h in 5% CO_2_ air. By the last 4 h, 10 μL MTT (3-(4,5-dimethyl-2-thiazolyl)-2,5-diphenyl-2-H-tetrazolium bromide) was added to each well and the supernatant was discarded after centrifugation. Dimethyl sulfoxide (DMSO) was added to a 24-well plate and oscillated for 10 min. Optical density (OD) values were tested in the microplate reader at the 570 nm wavelength, and the reported results were the average of 5 technical replicates.

### Statistical analysis

All data were analyzed using SAS statistical software (version 9.2) and they were expressed as mean ± SD. Significances among groups were tested using One-way ANOVA and the Wilcoxon rank-sum test. Differences were indicated statistically significant when *P*< 0.05.

## Results and Discussion

### Analysis of *L*. *plantarum* EPS

**Fig 1:**
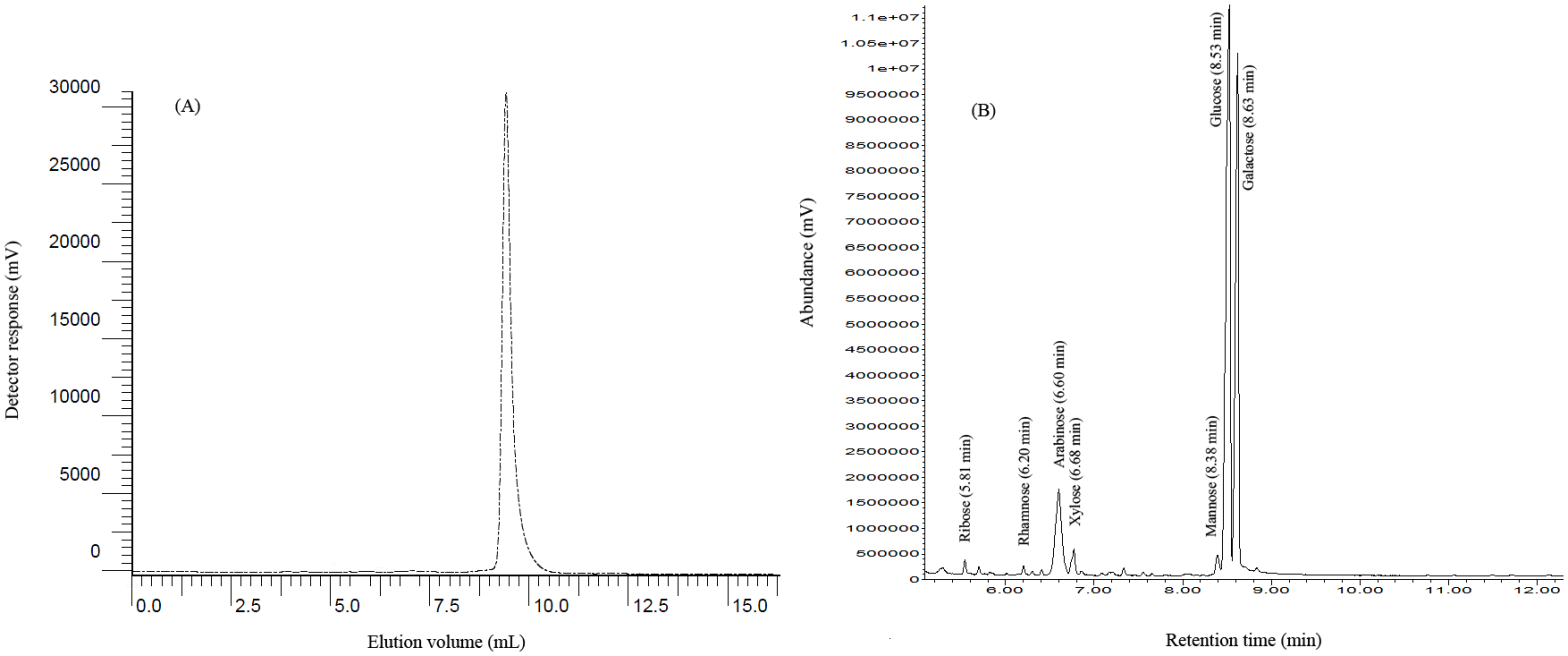
Homogeneity and Mw of the EPS by SEC analysis (A) and composition of the EPS by GC analysis (B).

The homogeneity, Mw and composition of *L*. *plantarum* EPS lyophilized powder were analyzed (Fig 1). Its purity was about 97%. The SEC profile of the EPS displayed a single and symmetrical peak, indicating homogeneity of *L*. *plantarum* EPS. The EPS was composed of ribose, rhamnose, arabinose, xylose, mannose, glucose and galactose with a molar ratio of 2:1:1:10:4:205:215, respectively (Fig 1). The molecular weight was approximately 2.4×10^6^ Da. There were little amounts of protein (28 μg/g) and endotoxins (0.20 EU/g) in the powder (S1 Table). The content of endotoxins were lower than that reported by Sheu et al [17], so any regulatory effect of EPS was not due to little amount of endotoxins.

### Cytokines production in blood serum and intestinal fluid of BALB/c mice

#### Nitric oxide production

**Fig 2:**
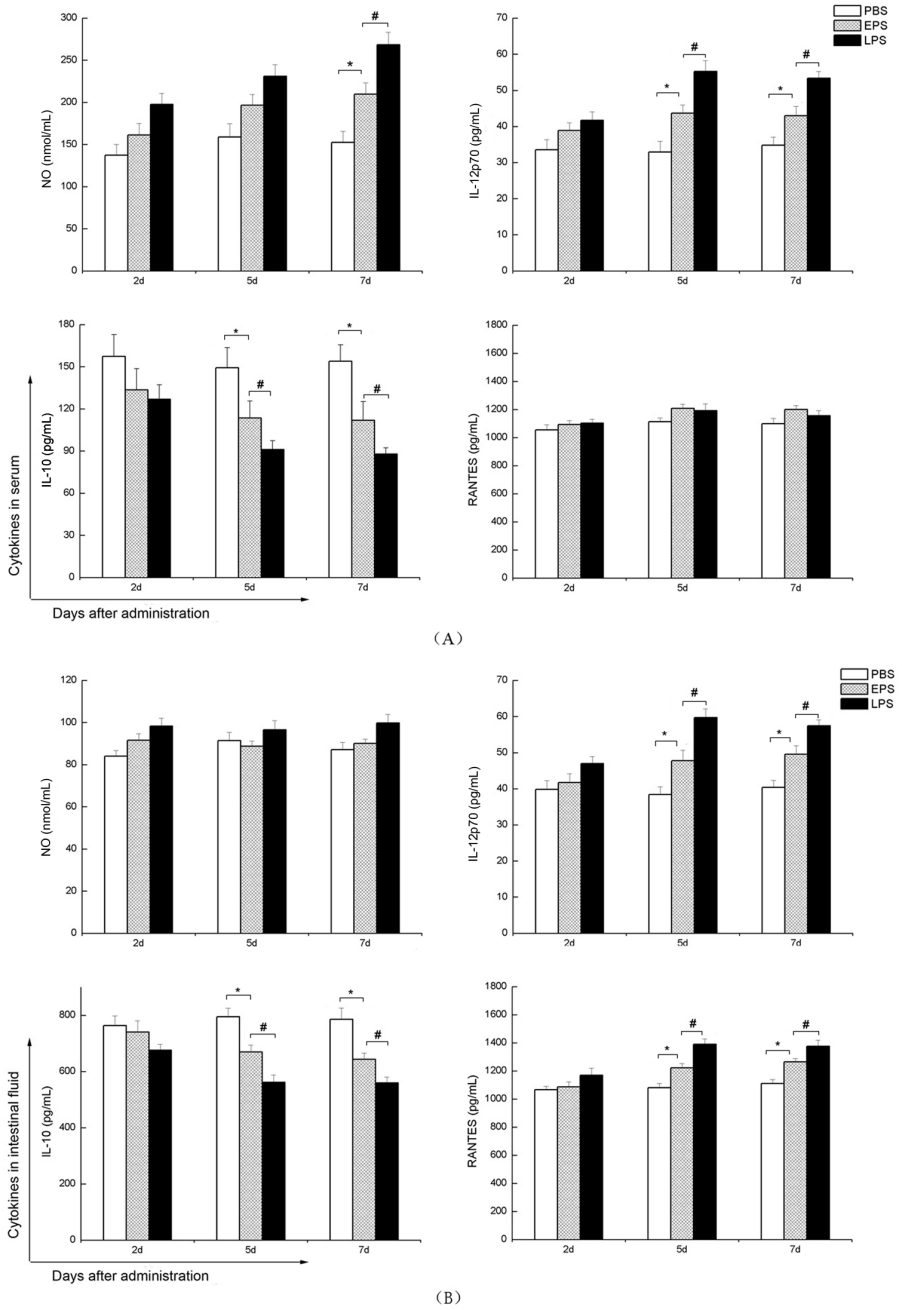
Production of NO, IL-12p70, IL-10 and RANTES in serum (A) and intestinal fluid (B) of BALB/c mice treated with EPS and LPS. Concentration of NO, IL-12p70, IL-10 and RANTES in the serum or intestinal fluid of mice that received *L*. *plantarum* EPS for 2, 5 and 7 days. All values were means ± SD (n = 12). Statistically significant differences are indicated as follows:*P< 0.05 versus PBS group, ^#^ *P*< 0.05 for EPS group versus LPS group.

NO is one of the most important bioactive substances in the immune system, and is generated by immune system cells such as DCs [23]. As an intracellular messenger molecule it mediates a lot of biological functions and especially participates in activating macrophages [24]. The production of NO secreted in the serum and the intestinal fluid in treated mice by EPS or LPS is shown in Fig 2. There was an increase of NO production in the serum of mice for the 7-day period of *L*. *plantarum* EPS administration (*P*< 0.05) and no significant increase of NO production for all treatment of period of assessed in the fluid of mice that received *L*. *plantarum* EPS. It also showed NO production was lower in the EPS group compared to the LPS group (*P*< 0.05).

#### IL-12p70 and IL-10 production

IL-12p70 is a critical cytokine needed for the differentiation of the initial T cell differentiation into Th (T helper) 1 cells [25], while IL-10 maybe contribute to the differentiation of Th 2 cells [26]. After the mice were gavaged with *L*. *plantarum* EPS, the production of IL-12p70 and IL-10 in the serum and the intestinal fluid is shown in Fig 2. There was a significant increase of IL-12p70 production and a decrease of IL-10 production in the serum or the fluid of mice that received *L*. *plantarum* EPS for the 5-day and 7-day period of *L*. *plantarum* EPS administration (*P*< 0.05).

IL-12p70 production in the serum or the intestinal fluid of EPS group was higher than that in PBS group, while IL-10 production was lower than that in the PBS group. This indicated that EPS induced DCs in LP of intestine to produce more IL-12p70 and less IL-10. As an endotoxin LPS can cause a strong immune response or damage. Compared to LPS group, lower changes of IL-12p70 and IL-10 production in EPS group did not cause a strong immune response or damage.

#### RANTES production

RANTES is secreted by ectopic endometrial cells, such as DCs, and activated T cells, causing the aggregation of more macrophages and T cells, and thus the secretion of more cytokines and chemokines [27, 28]. Concentrations of RANTES in the serum and the intestinal fluid after the mice were gavaged are shown in Fig 2. RANTES production increased significantly compared to PBS group in the fluid of mice that received *L*. *plantarum* EPS for the 5-day and 7-day period of *L*. *plantarum* EPS administration but lower than that in LPS group (*P*< 0.05) while there were no changes in the serum at all-time points of EPS administration. It was indicated that the intestine was the main interact site for EPS cross talk with DCs.

In this study, *L*. *plantarum* EPS had a great impact on the production of NO, IL-12p70, IL-10 and RANTES related to DCs in the serum and the intestinal fluid of BALB/c mice, which may result in activation of macrophages and inducing differentiation of Th0 cells to Thl cells, especially enhancing intestinal mucosal immunity. LPS is a common endotoxin that produces a strong immune response or damage in the body and is often designed as positive control in studies [17,18]. Our results showed that *L*. *plantarum* EPS had less impact on chemokine production than LPS, indicating that the EPS modulated a moderate immune response. Subsequently, we performed the *in vitro* experiment about the maturation of DCs treated by the EPS in order to understand the change of these cytokines production in the serum and the intestinal fluid.

### Phenotype and cytokine production of mouse BMDCs

#### DC surface markers expression

After rGM-CSF and rIL-4 treatment, bone marrow hematopoietic cells differentiated into immature BMDCs. Approximately 80% of the collected cell expressed the characteristic mouse DC surface marker, CD11c, by flow cytometer analysis (S1 Fig).

**Fig 3:**
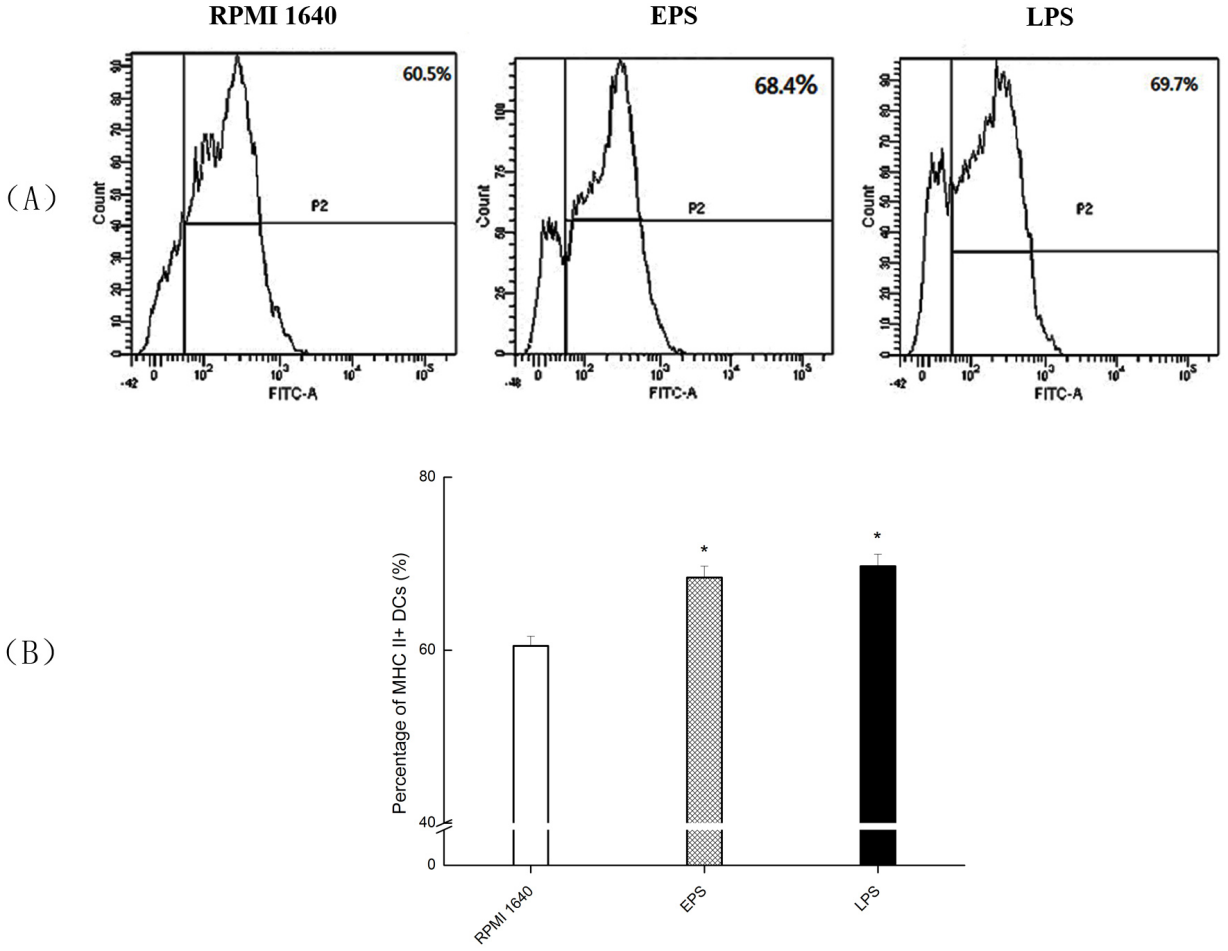
Expression of MHC II on BMDCs surface treated by EPS or LPS using Flow cytometer. (A) DCs were treated to a final concentration of 0 (RPMI 1640), 100 μg/mL EPS and 1 μg/mL LPS, respectively. (B) The expression levels of MHC II were statistical analyzed. All values were means ± SD (n = 3). Statistically significant differences are indicated as follows:*P< 0.05 versus RPMI 1640 group.

MHC II and costimulatory molecule CD86, both necessary in signal transduction for DC to recognize and uptake antigens, are the best indicators of DC immune function. The results are shown in Fig 3. MHC II expression of DCs treated with RPMI 1640 was 60.5%, while the group treated with 100 μg/mL EPS and LPS were 68.4% and 69.7%, respectively. Results showed that EPS could increase DCs surface MHC II molecule expression, and there was lower expression in the EPS group than the expression in the LPS group.

**Fig 4:**
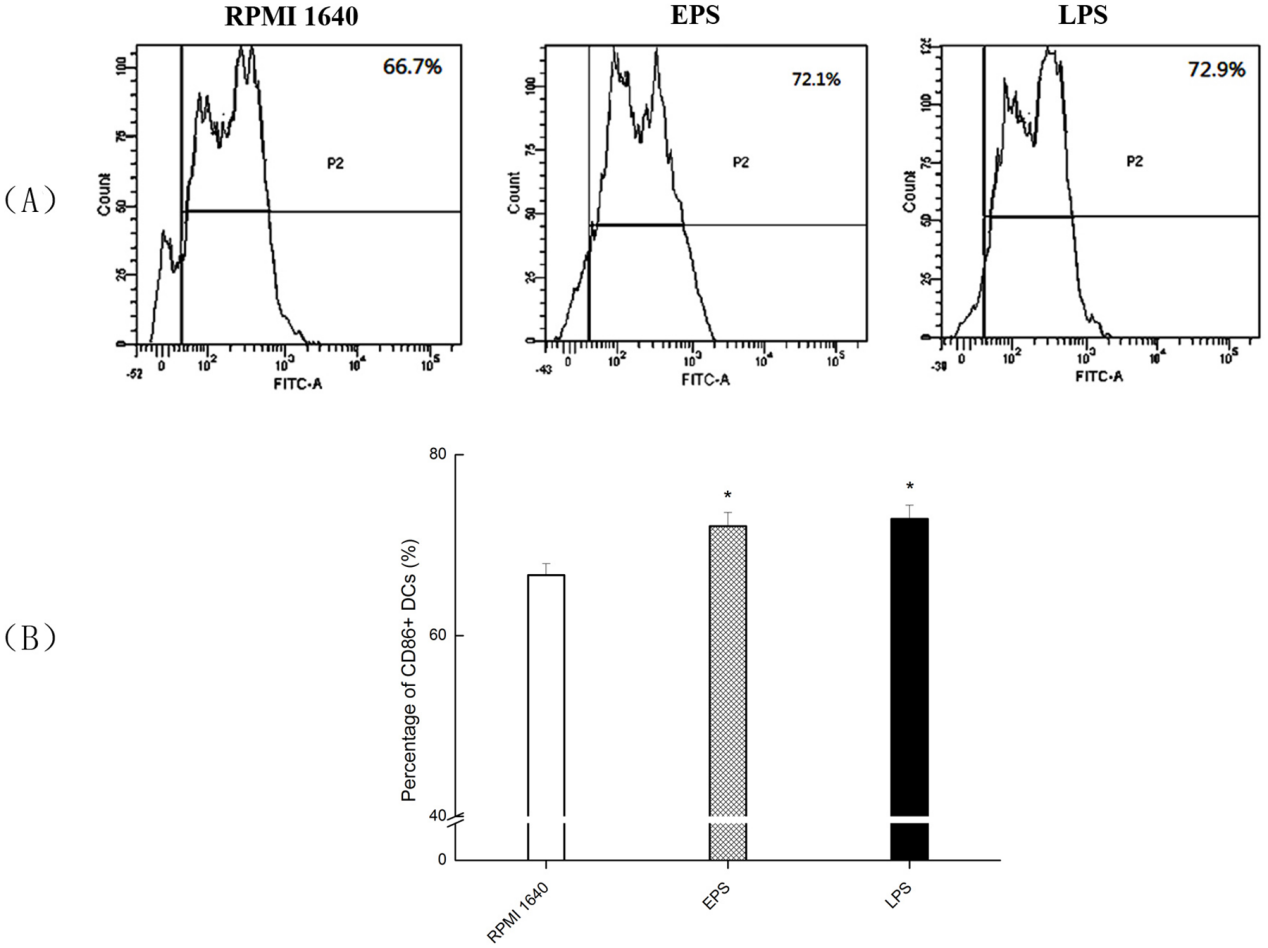
Expression of CD86 on BMDCs treated with EPS using flow cytometer. (A) DCs were treated to a final concentration of 0 (RPMI 1640), 100 μg/mL EPS and 1 μg/mL LPS, respectively. (B) The expression levels of CD86 were statistical analyzed. All values were means ± SD (n = 3). Statistically significant differences are indicated as follows:*P < 0.05 versus RPMI 1640 group.

CD86 expression was 66.7% in the RPMI 1640 group, 72.1% in the 100 μg/mL EPS group, and 72.9% in the LPS group (Fig 4). The results showed that EPS could significantly improve the expression of DCs costimulatory molecule CD86 (*P*< 0.05), not as much as that in LPS group.

As immune cells, DCs are the most powerful antigen presenting cells (APCs), and their function is related to their maturity [29]. Mature DCs can express a great amount of surface costimulatory molecules such as CD40, CD80, CD86 and MHC II, while immature DCs express little of these molecules. According to these surface markers, DCs can be divided into different types, with mature and immature being two of the major groups [30]. MHC II molecules are very important to the classification of DCs [31]. EPS was proved to promote the expression of MHC II molecules of DCs, suggesting that it may also induce the maturation of DCs. CD80, CD86 and CD40 are signs of mature DCs, and play indispensable roles in stimulating the immune response. CD86 activates DCs and Th0 cells, while memory T cells provide costimulatory signals. T cell activation requires two stimuli: (1) the binding of the T cell receptor (TCR) with antigen peptide-MHC molecule complexes, and (2) the combination of APCs surface with co-stimulatory molecules located on TCRs (CD40 and CD40L, or the interaction between either CD80 and CD28 or CD86 and CD28). The T cell response is said to be in the anergic state if the T cell recognition is processed by antigen-containing APC cells without allowing for the co-stimulatory molecules to promote the auxiliary signal [32]. Our experimental results showed that EPS induced DCs to produce more MHC II and CD86, which probably involved in enhancing DCs antigen presentation ability and in turn promoting the proliferation of T cells.

#### Nitric oxide production

**Fig 5:**
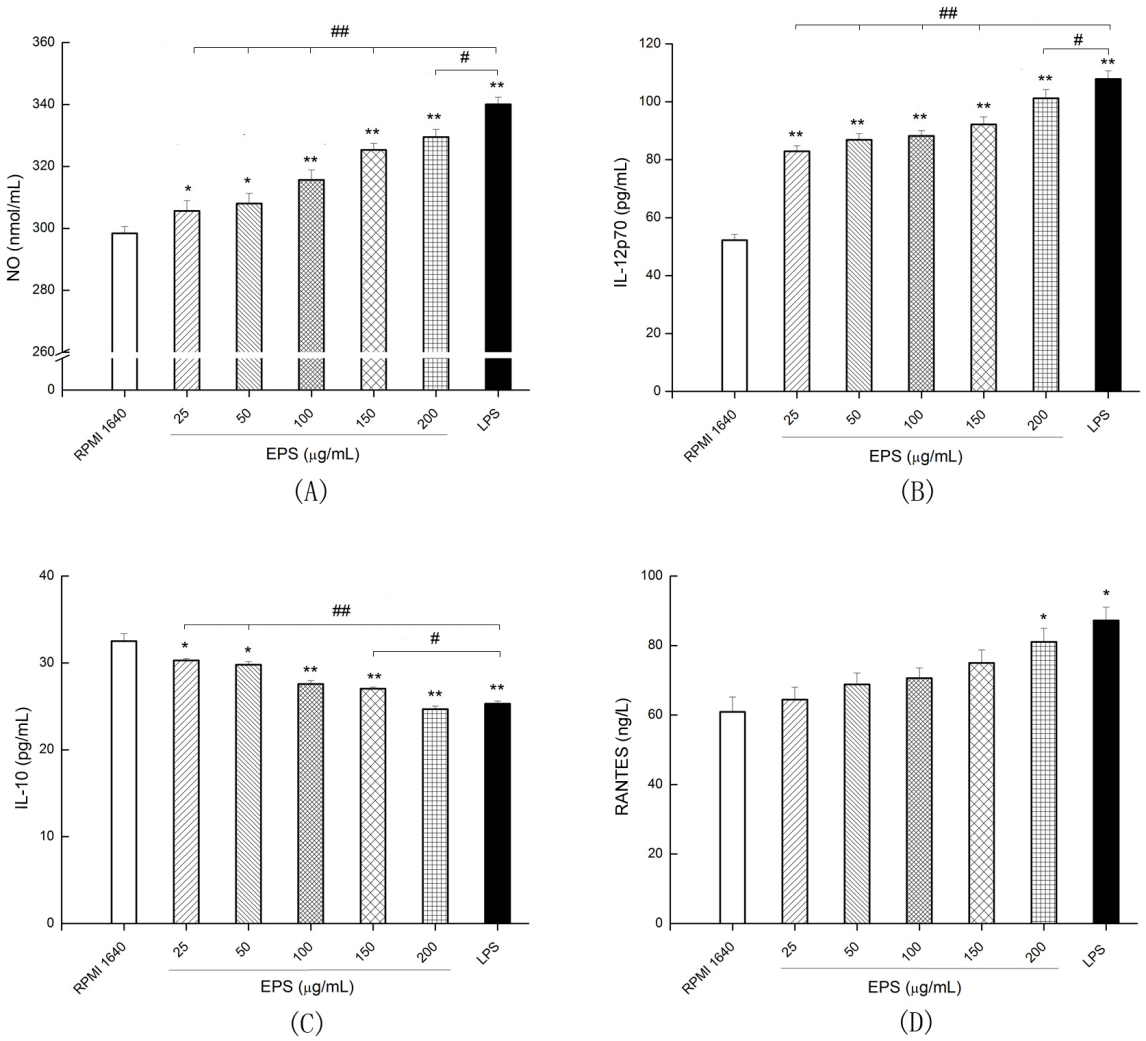
Production of cytokine NO (A), IL-12p70 (B), IL-10 (C) and RANTES (D) in culture supernatants of DCs treated with EPS and LPS. The EPS was added to a final concentration of 0 (RPMI 1640), 25, 50, 100, 150 and 200 μg/mL, respectively, and LPS was added to a final concentration of 1 μg/mL. After DCs were incubated at 37°C and 5% CO_2_ for 24 h, the supernatant was collected to determine NO, IL-12p70, IL-10 and RANTES concentration with an enzyme-linked immunosorbent assay (ELISA). All values were means ± SD (n = 3). Statistically significant differences are indicated as follows: *P< 0.05, **P< 0.01 VS RPMI 1640 group; ^#^ *P*< 0.05, ^##^ *P*< 0.01 for EPS group VS LPS group.

The quantity of NO secreted by EPS and LPS-treated DCs is shown in Fig 5(A). NO secretions from DCs treated for 24 h with EPS and LPS were all increased compared with NO secretions from the RPMI 1640 group. The result showed that NO secretion was 298.38 ± 1.13, 308.03 ± 0.28, 315.63 ± 0.20, 329.52 ± 0.45, and 340.03 ± 2.32 nmol/mL for the 0 (RPMI 1640), 50, 100, and 200 μg/mL EPS and LPS-treated DCs groups, respectively. EPS stimulated DCs to secrete NO, but secretions were lower in the EPS-treated group than the LPS-treated group.

#### IL-12p70 and IL-10 production

As shown in Fig 5(B), IL-12p70 secretion in the RPMI 1640 group was 52.25 ± 2.00 pg/mL. In 25, 50, 100, 150 and 200 μg/mL EPS and LPS groups, IL-12p70 secretion was 82.92 ± 1.89 pg/mL, 86.92 ± 2.08 pg/mL, 88.25 ± 1.80 pg/mL, 92.25 ± 2.50 pg/mL, 101.25 ± 3.00 pg/mL and 107.92 ± 2.75 pg/mL, respectively. These results showed that immature DCs treated with EPS could promote the secretion of T IL-12p70 (*P*< 0.01), but less than that in LPS group. As shown in Fig 5(C), EPS decreased DCs secretion of IL-10 compared with RPMI 1640 group (*P*< 0.01). Increase in IL-12p70 secretion and decrease in IL-10 secretion in EPS group were lower than that in LPS group.

Th0 cells play an important role in the immune response by secreting different ratios of Th1- and Th2-type cytokines. Th1-type cytokines such as IFN-γ and IL-2 play a role in phagocytosis by activating cytotoxic properties of effector cells. Th2-type cytokines such as IL-4 and IL-10 can stimulate B cells to produce antibodies and DCs also can interact with B cells to promote Th2 response [33]. DCs regulate differentiation of Th0 cells into Th1/Th2 cells through secretion of cytokines by DCs and T cells in order to maintain a balance between the two types of cells [34]. Thl and Th2 cell-mediated responses not only play different roles in the body's defense mechanism, but are also involved in differing immune pathology. Continually strong Thl cell immune response may lead to some organ-specific autoimmune diseases such as experimental allergic encephalomyelitis (experimental autoimmune encephalomyelitis, EAE) [35], Type I diabetes [36] and so on. Injecting the body with a special peptide specific to diabetes and DCs cultivated *in vitro* can effectively prevent the onset of diabetes. This is because when the peptide meets with the DCs, it can induce the expansion of Th2 cells, which are required for the peptide to induce a shift in the Th1 to Th2 immune response. The polysaccharide isolated from the cell walls of seaweeds has been proven to have potential clinical applications in the treatment of autoimmune diseases by keeping immune homeostasis [37]. In the treatment of autoimmune diseases, DCs are recognized as a great value immune cells [38]. DCs from different sources can cause different types of T cell responses, which can be divided into myeloid (DC1) and lymphoid (DC2) categories according to sources, phenotype and cytokine. DC1 induces Th0 cells to Thl-type cells, while DC2 induces Th0 cells to Th2-type cells [39]. However, some research found that myeloid DCs tended to induce Th2-type response, and lymphoid DCs tended to Th1-type response [25]. In a mouse transplantation model, liver DCs induced Th2-type response, and bone marrow DCs induced Th1-type cytokines such as IFN-γ production [40]; Compared with spleen DCs, human intestinal mucosal Peyer’s lymph node DCs strongly stimulate T cell activation and produce Th2-type cytokines, but Thl-type cytokines are only weakly induced [41]. Regulation of different types of DCs on Th1 and Th2 response were sometimes different probably because of species differences between humans and mice, or methods of DC purification and culture *in vitro*. However, some researchers thought that this was because of differences in DC IL-12 secretion in various developmental stages [42]. IL-12 was a key member of the third DC signal in regulating Th1/Th2 response, and could determine the direction of Th0 cell differentiation [43]. IL-12 is critical to the differentiation of Th0 cells to Thl cells, while IL-4 tends to promote Th2-type cell response in general. Other cytokines in the surrounding environment also contribute to the development of DCs, for example, IFN-γ and IL-2 can induce immature DC to differentiate into DC1, which not only express costimulatory molecule CD86, but also secrete large amounts of IL-12, and promote the differentiation of Th0 cells to Thl cells. Th2 cytokines, such as PGE2, promote differentiation of immature DC to DC2. PGE2 can express a great amount of costimulatory molecules but little of IL-12, and can induce differentiation of Th0 cells to Th2 cells. IL-10, unlike PGE2, can inhibit the development of immature DCs in the early stage and keep DCs expressing costimulatory molecules and secreting IL-12 at a low level.

In this experiment, IL-12 secreted by EPS-treated DCs in supernatant was higher than that in the RPMI 1640 group. On the contrary, IL-10 secreted by EPS-treated DCs was lower than that in the RPMI 1640 group. There's a strong possibility that the EPS can induce differentiation of Th0 cells to Thl cells, and be able to induce the secretion of RANTES-Thl-type. Thus, the EPS has potential for inducing immune responses of Th0 cells to Thl-type cells and promoting innate immunity through the transition to adaptive immunity by regulating the types of cytokines and chemokines secreted by DCs.

#### RANTES production

Concentration of RANTES is shown in Fig 5(D). For treatment with 0 (RPMI 1640), 25, 50, 100, 150 and 200 μg/mL EPS, RANTES secretion was 60.91 ± 9.48 pg/mL, 64.46 ± 7.53 pg/mL, 68.80 ± 6.29 pg/mL, 70.62 ± 6.62 pg/mL, 74.99 ± 2.77 pg/mL and 81.05 ± 4.90 pg/mL, respectively. RANTES content in the 200 μg/mL EPS group was higher than that in the RPMI 1640 group (*P* < 0.05), but not as high as that in LPS group. Thus, 200 μg/mL EPS could promote DCs to secrete RANTES and could induce Thl-type based immune response and the transition of innate immunity to acquired immunity. RANTES plays a key role in migration and activation of leukocyte, resulting in the recruitment of CD4^+^ and CD8^+^ T cells to sites of inflammation [44], so these results suggested that EPS had the capability of inducing the migration and activation of Th1 cell by secreting RANTES of DCs treated with EPS.

#### Allogeneic lymphocyte proliferation

**Fig 6:**
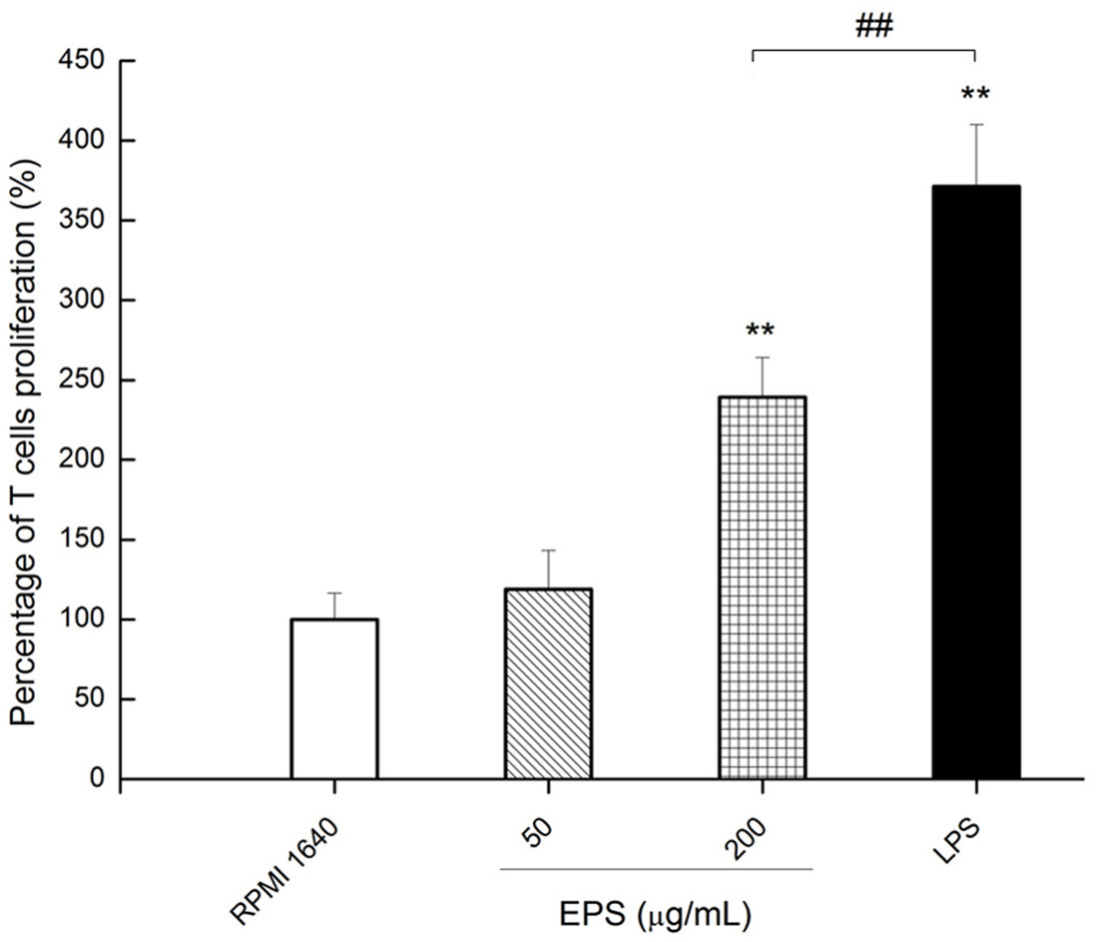
Effect of DCs treated with EPS on T cell proliferation. BMDCs were treated by 0 (RPMI 1640), 50, 200 μg/mL EPS and 1 μg/mL LPS, respectively. All values were means ± SD (n = 3). Statistically significant differences are indicated as follows:**P< 0.01 VS RPMI 1640 group; ^##^ *P*< 0.01 for EPS group VS LPS group.

EPS induced by stimulation of BMDCs allogeneic lymphocyte proliferation to reflect the impact of antigen-presenting ability of DCs. EPS concentration of 0 (RPMI 1640), 50, 200 μg/mL, and LPS induced lymphocyte proliferation rates were 99.99 ± 16.37%, 118.85 ± 24.13%, 239.34 ± 24.63% and 371.31 ± 38.65%, respectively (Fig 6). There was no significant increase in lymphocyte proliferation when treated with 50 μg/mL of EPS (*P*> 0.05) as compared with the RPMI 1640 group. However, when treated with the 200 μg/mL concentration of EPS, lymphocyte proliferation was significantly increased (*P*< 0.05), although it was still lower than that in the group treated with LPS (*P*< 0.01).

In this experiment T cells stimulated by DCs treated with EPS or LPS proliferated higher than the control group. This is because DCs in the RPMI 1640 group expressed a moderate level of MHC II molecules and low levels of CD86 and CD40 costimulatory molecules. As a result, T cell proliferation cannot be activated because of the lack of the necessary second signal. DCs treated with EPS or LPS became mature and provided T cells with the necessary surface molecules, resulting in proliferation of T cells.

At present, there are three signals involved in DC activating T cells to produce specific immune response: the first signal is the specific combination between the DC surface, MHC molecule/peptide complex and the Th cell surface TCR/CD3; the second signal is the interaction of coordinated stimulation molecules between the surface of DC and T cells [45]. These two signals together promote T cell activation and initiate the body's immune response. In this experiment, T cell proliferation was more highly stimulated by DCs treated with low or high concentration EPS than by DCs in the RPMI1640 group which were not treated with EPS. Without the treatment of EPS the DCs expressed MHC II and CD86 at a low level, so T cell proliferation cannot be activated. T cell proliferation was improved with EPS treatment due to maturation of the DCs resulting in adequate surface molecule expression. Kaliński proposed a third signal: at the start of immune response, DCs could express surface molecules selectively which could induce T cell polarization. Some of these molecules combined with the cell membrane, and some were soluble [42]. At the present time, IL-12 has been most studied, and it may balance the number of Thl and Th2, including regulatory T cells generation.

## Conclusions

Our study demonstrates that *L*. *plantarum* EPS has an immune modulation on DCs for the first time. We have shown that the EPS was homogeneous with Mw of 2.4×10^6^ Da. It increased NO production in the serum, enhanced IL-12p70 production in the serum as well as in the intestinal fluid, promoted RANTES production in the intestinal fluid and decreased IL-10 production both in the serum and in the intestinal fluid. *In vitro L*. *plantarum* EPS increased the production of NO, IL-12p70 and RANTES while reduced the secretion of IL-10. Furthermore, the EPS also up-regulated the expression of MHC II and CD86. Finally, DCs treated by the EPS promoted proliferation of T cells. *L*. *plantarum* EPS can promote maturation of DCs in BALB/c mice. We will further study the structural features of the polysaccharide and its molecular mechanism on modulation of immune function.

## Supporting Information

S1 Dataset Dataset. (XLSX)

S1 Fig The CD11c^+^ cells percentage analyzed by flow cytometer. After cultured with 20 ng/mL rGM-CSG and 20 ng/mL rIL-4, immature BMDCs were stained with either a PE conjugated isotype control or an anti-mouse CD11c monoantibody.
(TIFF)

S1 Table Composition of the EPS from *Lactobacillus plantarum*. (DOCX)

## Abbreviations

APC: antigen presenting cell
BMDC: bone-marrow-derived dendritic cell
CX3CR: CX3C-chemokine receptor
CX3CL: CX3C-chemokine ligand
DC: dendritic cell
EPS: exopolysaccharide
DMSO: Dimethyl sulfoxide
FBS: fetal bovine serum
IL: interleukin
L. plantarum: *Lactobacillus plantarum*
LAB: lactic acid bacteria
LP: lamina propria
LPS: lipopolysaccharide
MHC: major histocompatibility complex
MLN: mesenteric lymph node
MTT: 3-(4,5-dimethyl-2-thiazolyl)-2,5-diphenyl-2-H-tetrazolium bromide
Mw: molecular weight
NO: nitric oxide
PBS: phosphate buffer saline
PP: Peyer’s patch
RANTES: regulated upon activation in normal T-cell expressed and secreted
RPMIl640: RPMI l640 complete medium
SEC: size exclusion chromatography
SPF: specific-pathogen-free
TCR: T cell receptor
Th: T helper

## References

[1] de Vries MC, Vaughan EE, Kleerebezem M, de Vos WM. *Lactobacillus plantarum*—survival, functional and potential probiotic properties in the human intestinal tract. Int Dairy J. 2006; 16: 1018–1028. 10.1016/j.idairyj.2005.09.003

[2] Georgieva R, Iliev I, Haertlé T, Chobert J-M, Ivanova I, Danova S. Technological properties of candidate probiotic Lactobacillus plantarum strains. Int Dairy J. 2009; 19: 696–702. 10.1016/j.idairyj.2009.06.006

[3] Mathara J, Schillinger U, Kutima P, Mbugua S, Guigas C, Franz C, et al Functional properties of *Lactobacillus plantarum*strains isolated from Maasai traditional fermented milk products in Kenya. Curr Microbiol. 2008; 56: 315–321. 10.1007/s00284-007-9084-6 18175177

[4] Ismail B, Nampoothiri KM. Production, purification and structural characterization of an exopolysaccharide produced by a probiotic *Lactobacillus plantarum* MTCC 9510. Arch Microbiol. 2010; 192: 1049–1057. 10.1007/s00203-010-0636-y 20957348

[5] Garai-Ibabe G, Dueñas MT, Irastorza A, Sierra-Filardi E, Werning ML, López P, et al Naturally occurring 2-substituted (1,3)-β-d-glucan producing Lactobacillus suebicus and Pediococcus parvulus strains with potential utility in the production of functional foods. Bioresour Technol. 2010; 101: 9254–9263. 10.1016/j.biortech.2010.07.050 20691585

[6] Kim Y, Oh S, Kim SH. Released exopolysaccharide (r-EPS) produced from probiotic bacteria reduce biofilm formation of enterohemorrhagic Escherichia coli O157:H7. Biochem Biophys Res Commun. 2009; 379: 324–329. 10.1016/j.bbrc.2008.12.053 19103165

[7] Liu C, Lu J, Lu L, Liu Y, Wang F, Xiao M. Isolation, structural characterization and immunological activity of an exopolysaccharide produced by *Bacillus licheniformis* 8-37-0-1. Bioresour Technol. 2010; 101: 5528–5533. 10.1016/j.biortech.2010.01.151 20199860

[8] Pan D, Mei X. Antioxidant activity of an exopolysaccharide purified from *Lactococcus lactis subsp*. *lactis* 12. Carbohydr Polym. 2010; 80: 908–914. 10.1016/j.carbpol.2010.01.005 23911523

[9] Welman AD, Maddox IS. Exopolysaccharides from lactic acid bacteria: perspectives and challenges. Trend Biotechnol. 2003; 21: 269–274. 10.1016/S0167-7799(03)00107-0 12788547

[10] Blaschitz C, Raffatellu M. Th17 Cytokines and the gut mucosal barrier. J Clin Immunol. 2010; 30: 196–203. 10.1007/s10875-010-9368-7 20127275 PMC2842875

[11] de LeBlanc AdM, Dogi CA, Galdeano CM, Carmuega E, Weill R, Perdigón G. Effect of the administration of a fermented milk containing *Lactobacillus casei* DN-114001 on intestinal microbiota and gut associated immune cells of nursing mice and after weaning until immune maturity. BMC immunol. 2008; 9: 27 10.1186/1471-2172-9-27 18554392 PMC2459154

[12] Galdeano CM, Núñez IN, de LeBlanc AdM, Carmuega E, Weill R, Perdigón G. Impact of a probiotic fermented milk in the gut ecosystem and in the systemic immunity using a non-severe protein-energy-malnutrition model in mice. BMC gastroenterol. 2011; 11: 64 10.1186/1471-230X-11-64 21615956 PMC3125276

[13] Rescigno M, Urbano M, Valzasina B, Francolini M, Rotta G, Bonasio R, et al Dendritic cells express tight junction proteins and penetrate gut epithelial monolayers to sample bacteria. Nat immunol. 2001; 2: 361–367. 10.1038/86373 11276208

[14] Nicoletti C, Arques JL, Bertelli E. CX3CR1 is critical for Salmonella-induced migration of dendritic cells into the intestinal lumen. Gut Microbes. 2010; 1: 131–134. 21327020 10.4161/gmic.1.3.11711 PMC3023593

[15] Niess JH, Brand S, Gu X, Landsman L, Jung S, McCormick BA, et al CX3CR1-mediated dendritic cell access to the intestinal lumen and bacterial clearance. Science. 2005; 307: 254–258. 15653504 10.1126/science.1102901

[16] Lelouard H, Fallet M, de Bovis B, Méresse S, Gorvel JP. Peyer's Patch dendritic cells sample antigens by extending dendrites through M cell-specific transcellular pores. Gastroenterology. 2012; 142: 592–601.e593 10.1053/j.gastro.2011.11.039 22155637

[17] Sheu S-C, Lyu Y, Lee M-S, Cheng J-H. Immunomodulatory effects of polysaccharides isolated from *Hericium erinaceus* on dendritic cells. Process Biochem. 2013; 48: 1402–1408. 10.1016/j.procbio.2013.06.012

[18] Zhu J, Zhao L-H, Zhao X-P, Chen Z. *Lycium barbarum* polysaccharides regulate phenotypic and functional maturation of murine dendritic cells. Cell Biol Int. 2007; 31: 615–619. 10.1016/j.cellbi.2006.12.002 17289406

[19] e Sousa CR. Dendritic cells in a mature age. Nat Rev Immunol. 2006; 6: 476–483. 10.1038/nri1845 16691244

[20] Dubois M, Gilles K, Hamilton J, Rebers P, Smith F. A colorimetric method for the determination of sugars.Nature. 1951; 168: 167 10.1038/168167a0 14875032

[21] Nichols CM, Lardière SG, Bowman JP, Nichols PD, Gibson JAE, Guézennec J. Chemical characterization of exopolysaccharides from Antarctic marine bacteria. Microb Ecol. 2005; 49: 578–589. 10.1007/s00248-004-0093-8 16052372

[22] Inaba K, Inaba M, Romani N, Aya H, Deguchi M, Ikehara S, et al Generation of large numbers of dendritic cells from mouse bone marrow cultures supplemented with granulocyte/macrophage colony-stimulating factor. Journal Exp Med. 1992; 176: 1693–1702. 10.1084/jem.176.6.1693 1460426 PMC2119469

[23] Bogdan C. Nitric oxide and the immune response. Nat Immunol. 2001; 2: 907–916. 10.1038/ni1001-907 11577346

[24] Palmer RM, Ashton D, Moncada S. Vascular endothelial cells synthesize nitric oxide from L-arginine. Nature. 1988; 333: 664–666. 10.1038/333664a0 3131684

[25] Moser M, Murphy KM. Dendritic cell regulation of TH1-TH2 development. Nat Immunol. 2000; 1: 199–205. 10.1038/79734 10973276

[26] Reid SD, Penna G, Adorini L. The control of T cell responses by dendritic cell subsets. Curr Opin Immunol. 2000; 12: 114–121. 10.1016/S0952-7915(99)00059-X 10679408

[27] Appay V, Rowland-Jones SL. RANTES: a versatile and controversial chemokine. Trends Immunol. 2001; 22: 83–87. 10.1016/S1471-4906(00)01812-3 11286708

[28] Huang S-y, Xin H, Sun J, Li R, Zhang X-m, Zhao D. Estrogen receptor β agonist diarylpropionitrile inhibits lipopolysaccharide-induced regulated on activation normal T cell expressed and secreted (RANTES) production in macrophages by repressing nuclear factor κB activation. Fertil Steril. 2013; 100: 234–240. 10.1016/j.fertnstert.2013.02.052 23557759

[29] Adams S, O’Neill DW, Bhardwaj N. Recent advances in dendritic cell biology. J Clini Immunol. 2005; 25: 87–98. 10.1007/s10875-005-2814-2 15821885

[30] Wu L, Vremec D, Ardavin C, Winkel K, Süss G, Georgiou H, et al Mouse thymus dendritic cells: kinetics of development and changes in surface markers during maturation. Eur JImmunol. 1995; 25: 418–425. 10.1002/eji.1830250217 7875203

[31] Pieters J. MHC class II restricted antigen presentation. Curr Opin Immunology. 1997; 9: 89–96. Electronic identifier: 0952-7915-009-00089 10.1016/s0952-7915(97)80164-1 9039772

[32] Lu L, Qian S, Hershberger PA, Rudert WA, Lynch DH, Thomson AW. Fas ligand (CD95L) and B7 expression on dendritic cells provide counter-regulatory signals for T cell survival and proliferation. J Immunol. 1997; 158: 5676–5684. 9190916

[33] Maddur MS, Sharma M, Hegde P, Stephen-Victor E, Pulendran B, Kaveri SV, et al Human B cells induce dendritic cell maturation and favour Th2 polarization by inducing OX-40 ligand. Nat Commun. 2014; 5: 4092 10.1038/ncomms5092 24910129 PMC4388556

[34] Kadowaki N, Antonenko S, Lau JY-N, Liu Y-J. Natural interferon α/β–producing cells link innate and adaptive immunity. J Exp Med. 2000; 192: 219–226. 10.1084/jem.192.2.219 10899908 PMC2193254

[35] Serafini B, Columba-Cabezas S, Di Rosa F, Aloisi F. Intracerebral recruitment and maturation of dendritic cells in the onset and progression of experimental autoimmune encephalomyelitis. Am JPathol. 2000; 157: 1991–2002. 10.1016/S0002-9440(10)64838-9 11106572 PMC1885753

[36] Lee M, Kim A-Y, Kang Y. Defects in the differentiation and function of bone marrow-derived dendritic cells in nonobese diabetic mice. J Korean MedSci. 2000; 15: 217–223. PMC3054605 10.3346/jkms.2000.15.2.217 PMC3054605 10803701

[37] Hershkoviz R, Mor F, Miao HQ, Vlodavsky I, Lider O. Differential effects of polysulfated polysaccharide on experimental encephalomyelitis, proliferation of autoimmune T cells, and inhibition of heparanase activity. J Autoimmun. 1995; 8: 741–750. 10.1006/jaut.1995.0055 8579728

[38] Adikari SB. Cytokine-modulated dendritic cell immunotherapy in autoimmune diseases. Stockholm: Karolinska University Press; 2005

[39] de St Groth BF. The evolution of self -toleance: a new cell arises to meet the challenge of self-reactivity. Immunol Today. 1998; 19: 448–454. 10.1016/S0167-5699(98)01328-0 9785668

[40] Khanna A, Morelli AE, Zhong C, Takayama T, Lu L, Thomson AW. Effects of liver-derived dendritic cell progenitors on Th1-and Th2-like cytokine responses in vitro and in vivo. J Immunol. 2000; 164: 1346–1354. 10640749 10.4049/jimmunol.164.3.1346

[41] Iwasaki A, Kelsall BL. Freshly isolated Peyer's patch, but not spleen, dendritic cells produce interleukin 10 and induce the differentiation of T helper type 2 cells. J Exp Med. 1999; 190: 229–240. 10.1084/jem.190.2.229 10432286 PMC2195574

[42] Kaliński P, Hilkens CM, Wierenga EA, Kapsenberg ML. T-cell priming by type-1and type-2 polarized dendritic cells: the concept of a third signal. Immunol Today. 1999; 20: 561–567. 10.1016/S0167-5699(99)01547-9 10562707

[43] Snijders A, Kalinski P, Hilkens C, Kapsenberg ML. High-level IL-12 production by human dendritic cells requires two signals. Int Immunol.1998 10: 1593–1598. 10.1093/intimm/10.11.1593 9846688

[44] Vila-Coro AJ, Mellado M, de Ana AM, Martínez-A C, Rodríguez-Frade JM. Characterization of RANTES-and aminooxypentane-RANTES-triggered desensitization signals reveals differences in recruitment of the G protein-coupled receptor complex. J Immunol. 1999; 163: 3037–3044. 10477567

[45] Banchereau J, Steinman RM. Dendritic cells and the control of immunity. Nature. 1998; 392: 245–252. 10.1038/32588 9521319

