# Supplementary figures and images for: Exopolysaccharide Produced by Lactobacillus Plantarum Induces Maturation of Dendritic Cells in BALB/c Mice
Source: PLoS One. 2015 Nov 24;10(11):e0143743. doi: 10.1371/journal.pone.0143743 (PMC4658202; doi:10.1371/journal.pone.0143743)

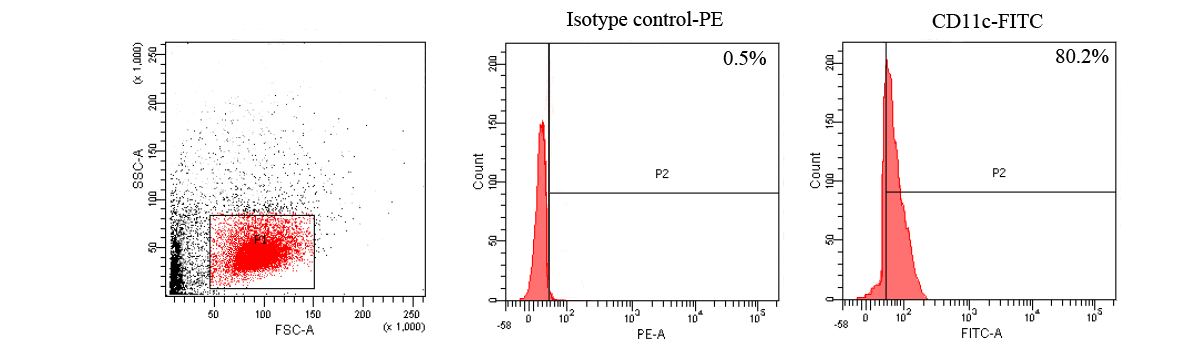

Supplement: S1 Fig — After cultured with 20 ng/mL rGM-CSG and 20 ng/mL rIL-4, immature BMDCs were stained with either a PE conjugated isotype control or an anti-mouse CD11c monoantibody. (TIFF) [file pone.0143743.s002.tiff]
