# Supplementary material for: Exopolysaccharide Produced by Lactobacillus Plantarum Induces Maturation of Dendritic Cells in BALB/c Mice
Source: PLoS One. 2015 Nov 24;10(11):e0143743. doi: 10.1371/journal.pone.0143743 (PMC4658202; doi:10.1371/journal.pone.0143743)
Supplement: S1 Table — (DOCX) [file pone.0143743.s003.docx]

**S1 Table Composition of the EPS from *Lactobacillus plantarum***

| Constituent | Content* |
| --- | --- |
| Total sugar | 970.82 ± 25.18 mg/g |
| Total protein | 28.00 ± 1.51 µg/g |
| Endotoxins | 0.20 ± 0.02 EU/g |

*The values are shown as mean ± SD (n=3)
